# Implementation of 3D spatial indexing and compression in a large-scale molecular dynamics simulation database for rapid atomic contact detection

**DOI:** 10.1186/1471-2105-12-334

**Published:** 2011-08-10

**Authors:** Rudesh D Toofanny, Andrew M Simms, David AC Beck, Valerie Daggett

**Affiliations:** 1 Department of Bioengineering, University of Washington, Box, 355013, Seattle, Washington, USA 98195-5013; 2 Biomedical and Health Informatics Program, University of Washington, Box, 355013, Seattle, Washington, USA 98195-5013; 3 eScience Institute, University of Washington, Box, 355013, Seattle, Washington, USA 98195-5013; 4 Department of Chemical Engineering, University of Washington, Box, 355013, Seattle, Washington, USA 98195-5013

## Abstract

**Background:**

Molecular dynamics (MD) simulations offer the ability to observe the dynamics and interactions of both whole macromolecules and individual atoms as a function of time. Taken in context with experimental data, atomic interactions from simulation provide insight into the mechanics of protein folding, dynamics, and function. The calculation of atomic interactions or contacts from an MD trajectory is computationally demanding and the work required grows exponentially with the size of the simulation system. We describe the implementation of a spatial indexing algorithm in our multi-terabyte MD simulation database that significantly reduces the run-time required for discovery of contacts. The approach is applied to the Dynameomics project data. Spatial indexing, also known as spatial hashing, is a method that divides the simulation space into regular sized bins and attributes an index to each bin. Since, the calculation of contacts is widely employed in the simulation field, we also use this as the basis for testing compression of data tables. We investigate the effects of compression of the trajectory coordinate tables with different options of data and index compression within MS SQL SERVER 2008.

**Results:**

Our implementation of spatial indexing speeds up the calculation of contacts over a 1 nanosecond (ns) simulation window by between 14% and 90% (i.e., 1.2 and 10.3 times faster). For a 'full' simulation trajectory (51 ns) spatial indexing reduces the calculation run-time between 31 and 81% (between 1.4 and 5.3 times faster). Compression resulted in reduced table sizes but resulted in no significant difference in the total execution time for neighbour discovery. The greatest compression (~36%) was achieved using page level compression on both the data and indexes.

**Conclusions:**

The spatial indexing scheme significantly decreases the time taken to calculate atomic contacts and could be applied to other multidimensional neighbor discovery problems. The speed up enables on-the-fly calculation and visualization of contacts and rapid cross simulation analysis for knowledge discovery. Using page compression for the atomic coordinate tables and indexes saves ~36% of disk space without any significant decrease in calculation time and should be considered for other non-transactional databases in MS SQL SERVER 2008.

## Background

Molecular dynamics (MD) simulations are routinely used to study the dynamic and structural properties of proteins and other macromolecules. MD simulations provide atomic-level resolution of a protein andits surrounding solvent environment as a function of time. There are no experimental techniques that can provide this level of detail. The direct results of an MD simulation are the coordinates of all atoms as a function of simulation time. Simulation time is divided into discrete time points or frames (akin to movie frames) that represent the coordinates for the entire system at that precise time. The assembled coordinate 'trajectories' (i.e. all frames) can be analysed for various factors and visualized to produce movies (examples of which can be found at http://www.dynameomics.org).

Nonbonded interactions within a protein are critical to its thermodynamic behaviour, contributing to packing and electrostatic energies reflected in the enthalpy. Such nonbonded interactions include but are not limited to hydrogen bonds, salt bridges, and hydrophobic contacts. Fluctuations in these nonbonded contacts as a function of time dictate dynamic behaviour and the conformations accessible to the protein. Dynamics are crucial for our understanding of protein function [1], folding and misfolding [2,3].

We have recently undertaken and completed a large scale project, Dynameomics, in which we have simulated the native states and unfolding pathways of representatives of essentially all autonomous protein fold families [4]. These fold families, or metafolds, were chosen based on a consensus between the SCOP, CATH and DALI domain dictionaries, which we call a consensus domain dictionary (CDD) [5,6]. For our recent release set [5] there are 807 metafolds, representing 95% of the known autonomous domains in the Protein Data Bank (PDB). The Dynameomics database represents the largest collection of protein simulations in the world and contains 10^4 ^more structures than the PDB.

The coordinates of the MD simulations and our set of standard analyses have been loaded into a relational database. This Dynameomics database is implemented using Microsoft SQL server with the Windows Server operating system (see [7] for a more detailed description). The Dynameomics protocol includes one native state simulation, and at least 5 thermal unfolding simulations, which can be used to characterize the unfolding process of the domains. In order to explore the dynamics and folding in these simulations we often calculate the nonbonded contacts for each frame of the simulation. This problem has been well studied and is also known as the nearest neighbor search problem [8]. The calculation is computationally expensive; as the naïve approach is to test all possible pairs of atoms in the system. The number of protein atoms or amino acids is often used as a proxy for the overall simulation size. The average number of protein atoms in the proteins in our Dynameomics set of simulations is 2150, with the smallest system consisting of 494 protein atoms and the largest of 6584 protein atoms. As all of the atoms in our simulations are in motion, all pairs of atoms need to be re-evaluated for each frame of the simulation, so in the case of a 51 ns native state simulation sampled at 1 picosecond (ps) resolution, we have 51,000 frames of pairs of contacts to evaluate. Calculating the nonbonded contacts without any acceleration method is not practical for a large number of simulations such as in a project like Dynameomics.

### Spatial indexing overview

Spatial indexing is a commonly used method by programmers of 3D video games, in which collision between objects are detected [9], though the methods date back further in molecular simulation [10,11] and other approaches similar in spirit have been described [12]. The basic approach based on the cell index method [10] is as follows: in order to accelerate the detection of near neighbour objects in 3D space, the space is split into relatively uniform small 3D bins. Each of the bins is given an index and the objects in the system are sorted into the indexed bins based on their 3D coordinates. Neighboring objects can then be detected by performing a distance calculation on all pairs of objects in the same or immediately adjacent neighboring bins. There are a number of other algorithms that could be used to speed up the discovery of nearest neighbors including B-trees, kd-trees, Z-order curves, Verlet neighbor lists, however, we decided to implement the cell index like method [10] since we already have experience in implementing this in our in-house MD simulation software and have found it to be very effecient.

**Figure 1:**
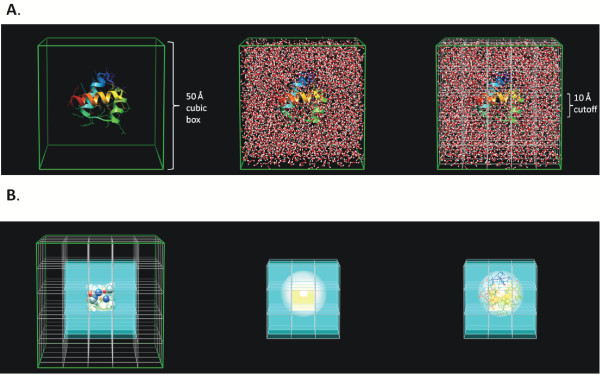
**Schematic showing the spatial binning of a periodic box and subsequent evaluation of atomic contacts in adjacent bins.** a). Schematic showing the spatial binning of a periodic box. In this example, the protein (1enh, the engrailed homeodomain) is simulated in a periodic box of water molecules with dimensions of 50 Å. The periodic box is split into smaller boxes of 10 Å these are the 3 dimensional bins. For clarity the boxes in this figure are 10 Å, in our implementation we use box dimensions of 5.4 Å. Each bin is assigned an index and hence every atom at every time point will have associated X, Y, Z coordinates and a bin index. b) Schematic describing the evaluation of adjacent bins. To reduce the computational expense for detecting nearest neighbors one evaluates the atomic pair distance for atoms that are within the same bin and the immediately adjacent bins, shown by the cyan boxes. The white transparent sphere has a radius represented by the cutoff distance used as our criteria for the consideration of an atom to be in contact with the atom at the center of the sphere.

Our MD simulation and analysis engine, *in lucem *molecular mechanics (*il*mm) [13] implements a spatial indexing (hashing) algorithm for rapid parallel calculation of nonbonded terms [14]. The Dynameomics simulations are performed in a periodic box of explicitly represented water molecules with the hydrated protein in the center of the box. This periodic box is conceptually similar to an orthorhombic unit cell in crystallography. We typically employ the NVE ensemble as the framework for our classical simulation where the number of atoms (N), periodic box volume (V), and total energy (E) are constant throughout the simulation. The fixed volume is achieved by using a fixed set of periodic box dimensions for the system. As such a system's spatial hash structure can be utilized unchanged throughout the simulation. To accommodate typical nonbonded interaction criteria we split our periodic box into bins of at least 5.4 Å on the three dimensions. This distance was chosen as it is the maximum distance we consider a pair of atoms to be in contact [15] (Figure 1A).

SQL Server 2008 supports two types of compression, which can be applied separately to the data and indices associated with a table (row and page level compression is only available in MS SQL server 2008). Row compression is a more efficient representation of row data; it involves storing fixed length columns in a manner similar to variable length columns where repeated bytes are compressed. For coordinate columns, which are a set of five 32 bit fixed length columns, the storage savings for row compression are small. Page compression, which is built on top of row compression, stores repeating values in a single structure for each page and then references that structure. This can result in significant savings as coordinate tables contain numerous columns with repeated data like atom number that are used for relational joins to retrieve atom information like name, mass, element.

## Results and Discussion

**Figure 2:**
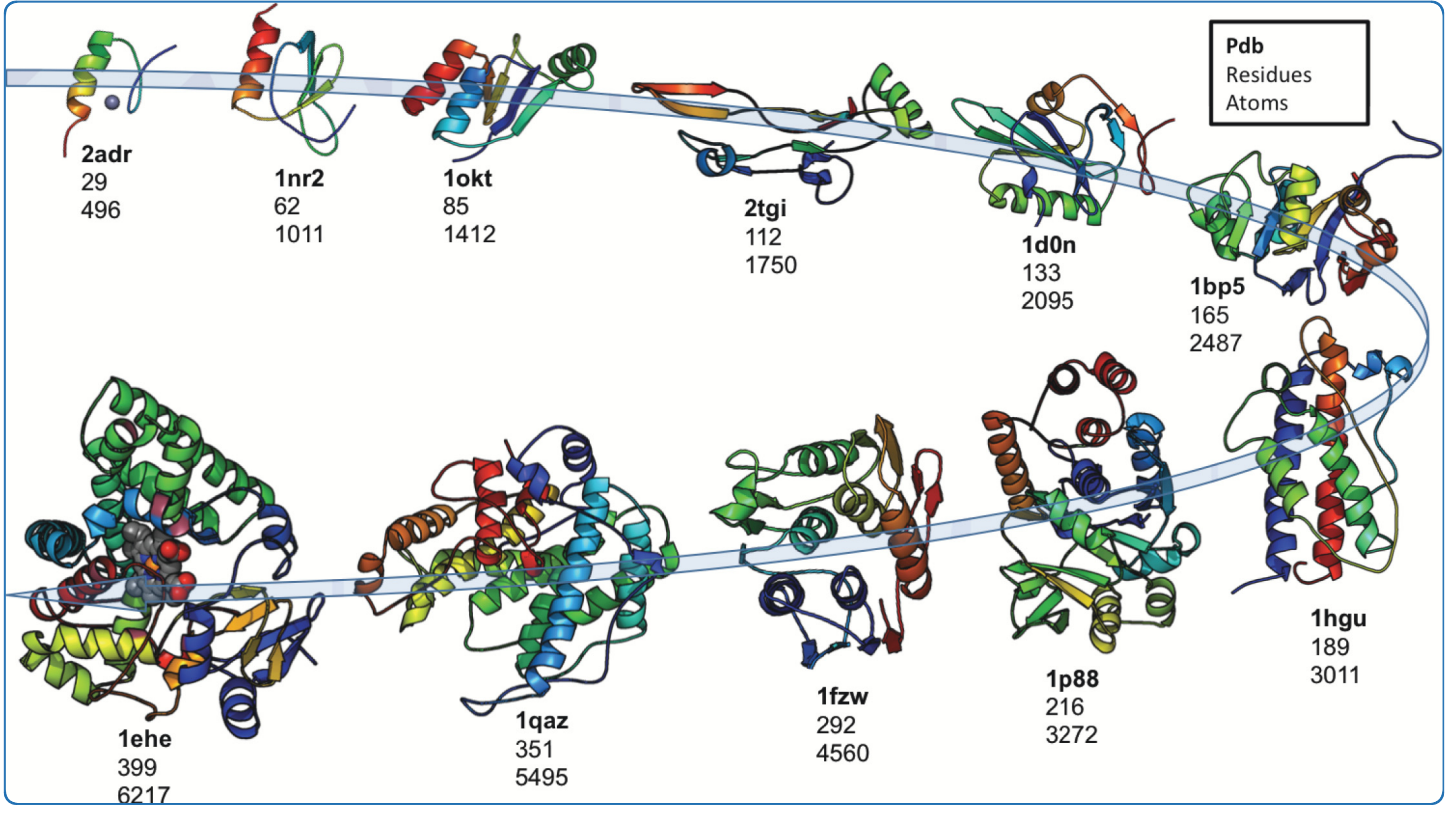
**11 metafolds representative of sequence length in Dynameomics**. The proteins are ordered by the number of amino acid residues in each protein. Also see, Table 1.

**Figure 3:**
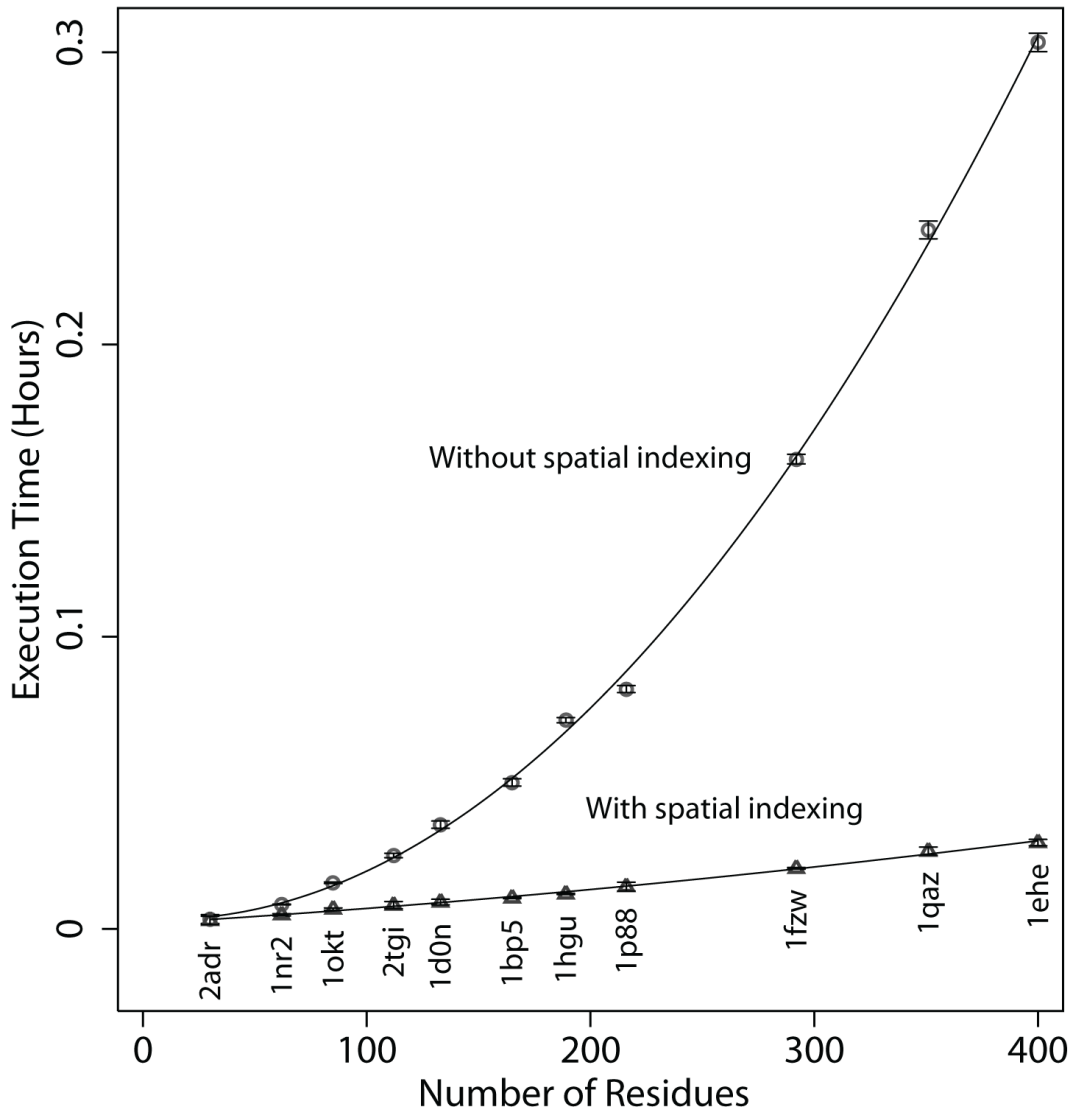
**Comparison of heavy-atom contacts query with and without spatial indexing for 11 metafold representatives over 1 ns**. With no spatial indexing (circles) applied the calculation of heavy-atom contacts over 1 ns (1000 frames) takes an average of ~20 minutes (n = 6) for the largest protein 1ehe. For the smallest protein, 2adr, the average time taken is around 10 seconds. When spatial indexing is applied (triangles) the is a dramatic decrease in execution time for 1ehe from ~20 minutes to an average of 1 minute 46 seconds. There is almost no change in execution time for 2adr since it is an extremely small protein, such that spatial indexing has little effect.

We investigated the effect of using spatial indexing in simulation coordinate tables to accelerate the discovery of nonbonded atomic contacts. We compared the execution times for the commonly employed heavy-atom (i.e. non-hydrogen) contact query for 1 ns (1000 frames) of each of our 11 representative metafolds selected from across the spectrum of system size (Figure 2, Table 1). In Figure 3, we show the average execution time for the 1 ns heavy-atom contact queries with and without the use of spatial indexing. Additional file 1, Table S1 presents a more detailed comparison of the execution times with and without the spatial index. The results show that for all 11 cases that we achieved between 13.8 and 90.3% decrease in execution time when using spatial indexing, i.e. the time to calculate contacts was between 1.2 and 10.3 times faster. As expected, query times decreased as the number of distance calculations is decreased (p < 0.05) for 11 metafolds. For one very small transcription factor metafold (PDB: 2ADR) the heavy-atom contact execution time did not significantly change when using spatial indexing. Further investigation of this case indicates that very small systems enjoy a much more limited decrease in run-time. Specifically, the transcription factor of 2ADR has a radius of gyration of 8.5 Å and as each spatial bin has the minimum dimensions of 5.4 Å by 5.4 Å by 5.4 Å, the entire protein is covered by one 27-bin chunk. For such cases, the spatial index method reduces into the naïve neighbour discovery method in which all pairs of heavy-atoms are considered and there is no significant difference in run-time. However, it is worth noting that the run-time did not increase. Practically speaking, this means that we do not pay a penalty for the indexing when it is not effective, and, as a result, all proteins may be treated identically, regardless of size.

**Table 1:** 11 representative proteins - number of residues and number of atoms

PDB4	Name	Residue Range	# residues	# protein atoms
2adr	Domain of Adr1 DBD from S. cerevisiae	102-130	29	496
1nr2	Thymus and activation-regulated chemokine	8-69	62	1011
1okt	Glutathione S-transferase	1-85	85	1412
2tgi	Domain of transforming growth factor-beta 2 (TGF- B2)	1-112	112	1750
1d0n	Horse plasma gelsolin	27-159	133	2095
1bp5	Domain of serum transferrin	82-246	165	2487
1hgu	Human growth hormone	2-190	189	3011
1p88	3-phosphoshikimate 1-carboxyvinyltransferase	25-240	216	3272
1fzw	Monomer of glucose-1-phosphate thymidylyltransferase	2-293	292	4560
1qaz	Alginate Lyase A1-III	4-354	351	5495
1ehe	Cytochrome P450nor	5-403	399	6217

The significant decrease in execution time for identification of nonbonded contacts had three significant implications. First, contact calculations are substantially more tractable for very large proteins in Dynameomics. Considering one of the largest fold representatives (1ehe) in our Dynameomics set, which contains 399 residues (plus a heme moiety), the average execution time dropped from 18 minutes and 10 seconds to just under 1 minute and 45 seconds. Second, the query execution time is fast enough to enable us to perform large-scale multi-simulation analyses. Dynameomics is really about the knowledge discovery over a large number of protein systems. For example, a key query for Dynameomics is to identify all of the types of hydrophobic contacts across the native state simulations for all of the 807 metafold representatives to identify patterns. Such an all-encompassing search is no longer impractical as contact queries across multiple servers can be executed to return the contact set rapidly. Third, as the calculation can be run in near real time, contact queries can be performed on the fly where the result set can be streamed through analyses rather than stored permanently and regenerated when required. The cost of disk space to save the contact results may exceed the size of the original coordinate data from which they were derived. Hence, we would need to more than double the size of our existing database configuration if we were to consider storing the result of contact queries for all simulations. Furthermore, the ability to run *ad-hoc *on-the-fly analyses is the heart of our exploratory mining efforts for Dynameomics. Our exploratory visualization tool for extremely large datasets, dubbed DIVE (Data Intensive Visualization Engine) can connect to our SQL database and rapidly visualize, and act upon, millions of data points in many dimensions such as the nonbonded contact queries [16].

**Figure 4:**
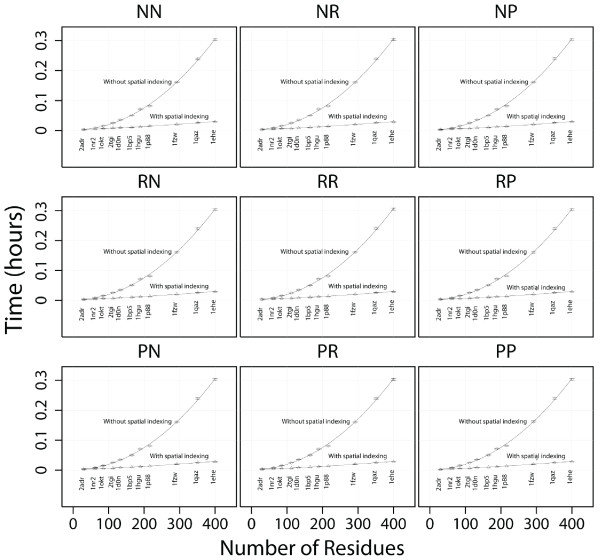
**Comparison of 9 sets of compression options and their effect on query execution times with and without spatial indexing**. P = page, R = row, N = none, e.g. PP represents Page compression on both the data and index where as NR represents no data compression but row compression on the index. Execution times with spatial indexing off are in circles and triangles denote spatial indexing on.

**Figure 5:**
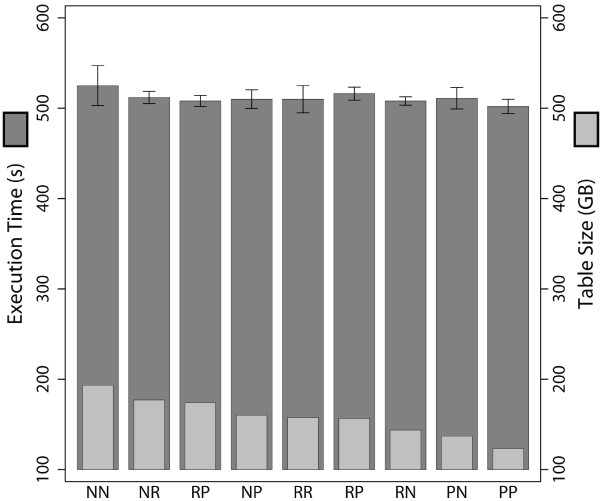
**Total table size using different compression options and total execution times for the 1 ns contacts query**. P = page, R = row, N = none, e.g. PP represents Page compression on both the data and index where as NR represents no data compression but row compression on the index. Total execution times are the sum of the individual representatives query times. Even with the largest compression using page compression on both the data and indexes, total execution times were comparable to the non-compressed tables.

The heavy-atom contact query is a computationally expensive calculation that queries the atomic coordinate tables (the largest tables in our database). We decided to use this query (under spatial indexing conditions) as the basis for testing different options of data and index compression. The goal of this aspect of the study was to find a data and index compression scheme for the coordinate tables that saves disk space but does not significantly affect query execution times. We investigated 9 sets of data and index compression options and applied each of these to each of our 11 metafold representative coordinate tables. As an initial test, we calculated the heavy-atom contacts for the first nanosecond of each metafold's trajectory with each set of compression options for the coordinates table. Figure 4 shows the average execution times for calculating heavy-atom contacts (with and without spatial indexing) for 1 ns (i.e. 1000 frames) for each metafold with every type of compression. Figure 5 compares the average compression for each set of data and index compression options across all the 11 metafolds. The average compression ranges from 8 - 36%. The lowest level of compression arises from having no data compression but row compression on the index. The largest level of compression was obtained using page compression on both the data and indexes.

**Figure 6:**
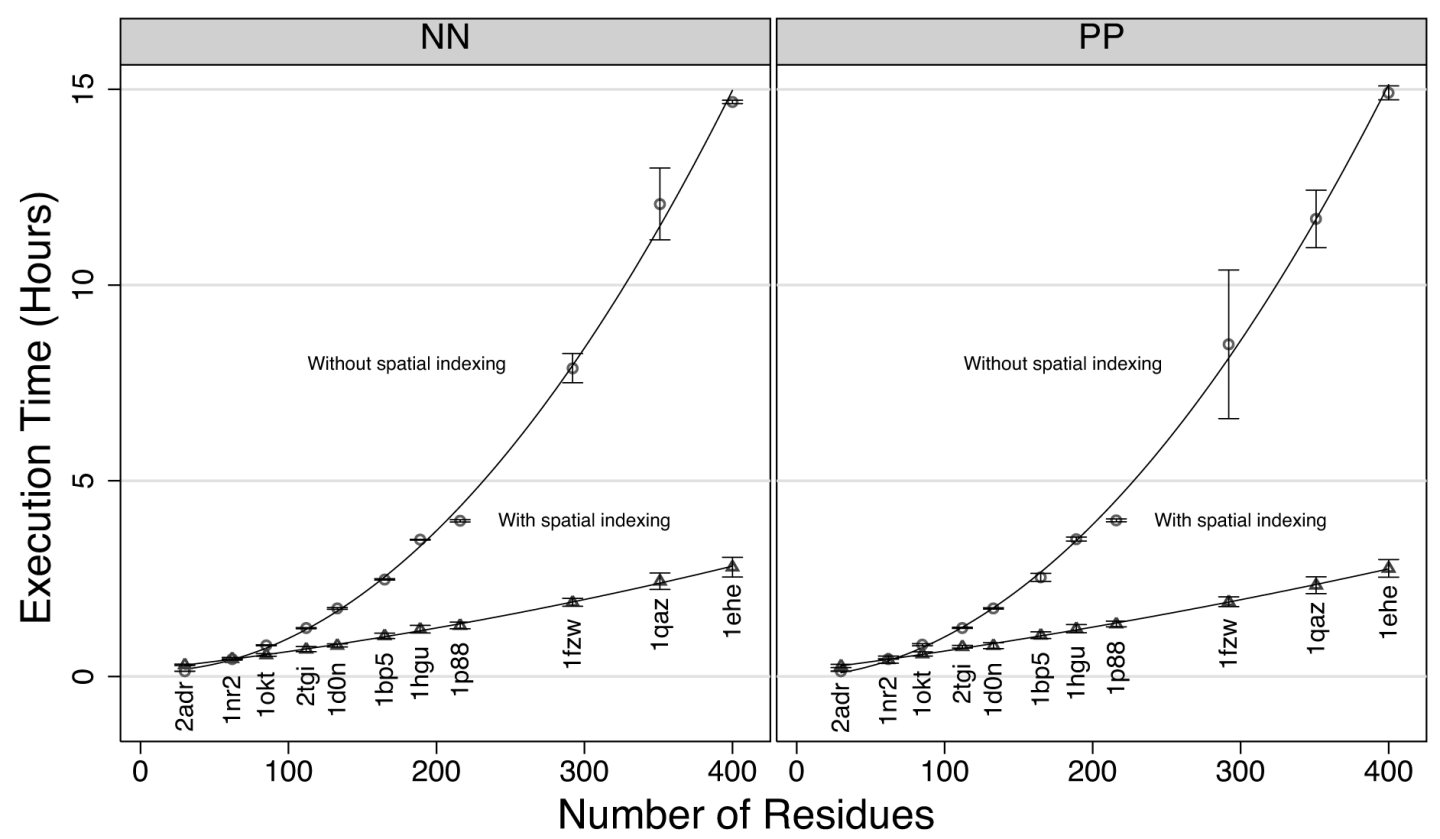
**Comparison of heavy-atom query execution times with and without spatial indexing using uncompressed tables and page/page compression**. NN denotes no data and no index compression whilst PP denotes page compression on the data and index. There is no significant difference in execution times.

Comparing the total execution times (Figure 5) for the heavy-atom contacts over 1 ns on the compressed tables versus the non-compressed tables all sets of compression options lie within the standard deviation of the total execution times for the non-compressed tables. This result is significant as it indicates that we can adopt any of the compression sets and retain the same fast execution time for the heavy-atom contacts query. The prospect of being able to compress our coordinate data by 36%, using both page data and page index compression, was investigated further by calculating heavy-atom contacts for the full 51 ns (i.e., 51,000 frames) of each simulation. We compared the execution times (with and without spatial indexing) of the non-compressed coordinate tables to that of the 36% compressed data (Figure 6). There is no significant difference (Additional file 2, Table S2a, b) in execution times for examining the full trajectories when calculating contacts from an uncompressed coordinate table and page compression on coordinate tables and indexes with and without spatial indexing. This result confirms our earlier finding on uncompressed tables and demonstrates that the problem scales appropriately (Figure 5). Additional file 3, Table S3a, b compares the full trajectory execution times for the compressed and non-compressed tables with and without spatial indexing. Spatial indexing, both with and without compression, is 1.4 to ~5.3 times faster for all but the smallest two proteins. As 85% of the Dynameomics database is made up of atomic coordinate simulation data, a 36% space saving of 70 TB of uncompressed data will net an additional 21 TB of disk space. This compression scheme is oriented towards repeated values, such as those found in dimension keys.

These data suggest that for other static (i.e. non-transactional) databases implemented in MS SQL Server 2008, compression may offer substantial disk savings. Furthermore, the framework of spatial indexing in a SQL database to speed up the discovery of near neighbours can be applied to other neighbor discovery problems, such as calculation of the distance between galaxies and/or planets in the field of astrophysics. The spatial indexing framework can be applied to those problems where the space is not bound to three-dimensions, but have fixed dimensional boundaries, and could be used to cluster highly dimensional data sets.

## Conclusions

The spatial indexing implementation presented herein for our multi-terabyte MD simulation database decreases neighbour discovery and interaction query execution times by up to 90%. While the speed-up for small proteins was less pronounced, the implementation was suitable for all sizes of simulation systems without introducing overhead for small systems and significant improvement in performance for larger systems. In addition, this work shows that all sets of page and row compression across the data and indexes we tested have no appreciable effect on the run-time of the heavy-atom contact query. The page/page compression set for data in indexes yielded a 36% disk savings for full trajectories over non-compressed tables. This represents a huge savings for large data sets.

## Methods

### MD Simulations

Details of how we selected the 807 metafolds for simulation in our Dynameomics project can be found elsewhere ([4,5]). The MD simulations were performed using *in lucem *molecular mechanics (*il*mm) [13] following the Dynameomics protocol described by Beck et al. [15]. Each of the metafolds had at least one native-state simulation performed at 298 K for at least 51 ns of simulation time, along with 5-8 unfolding simulations at 498 K with two of these simulations being at least 51 ns long. Structures were saved every 0.2 ps for the shorter simulations and every 1 ps for the longer simulations. Coordinates and analyses from the simulations were loaded into our Dynameomics database (for a more in-depth discussion on the development and technical details of the database see [7]).

When a simulation is loaded into the database, it is assigned a unique identifier and a specific location, i.e. server and database. Three tables were created in the assigned database to hold the underlying data for the simulation: a trajectory coordinate table, a box table, and bins table. Each table was named by the simulation identifier, for example the tables for simulation with identifier 37 would be "Coord_37," "Box_37," and "Bins_37." The coordinate table contained columns for each of the three-dimensional coordinates, atom number, step, structure identifier, and instance (which is used to identify monomers in a multimer system). The box table had columns for the x, y, and z dimensions of the periodic box at each time point. The bins table recorded the set of adjacent bins for each primary bin in the box. All three tables had clustered primary keys and constraints and the coordinate table also had a secondary covering index.

We selected 11 metafolds to represent the range in sequence size that our Dynameomics project covers from the smallest: ADR1 DNA-binding domain from *Saccharomyces Cerevisiae *(2adr, 29 residues and a zinc ion, [17]); to one of our largest: cytochrome P450 (1ehe, 399 residues and heme, [18]). Figure 2 shows the metafolds selected. In the test conducted in this study we chose to look at the 51 ns native state (298 K) simulations for each of these proteins.

### Implementation of spatial indexing in the database

**Figure 7:**
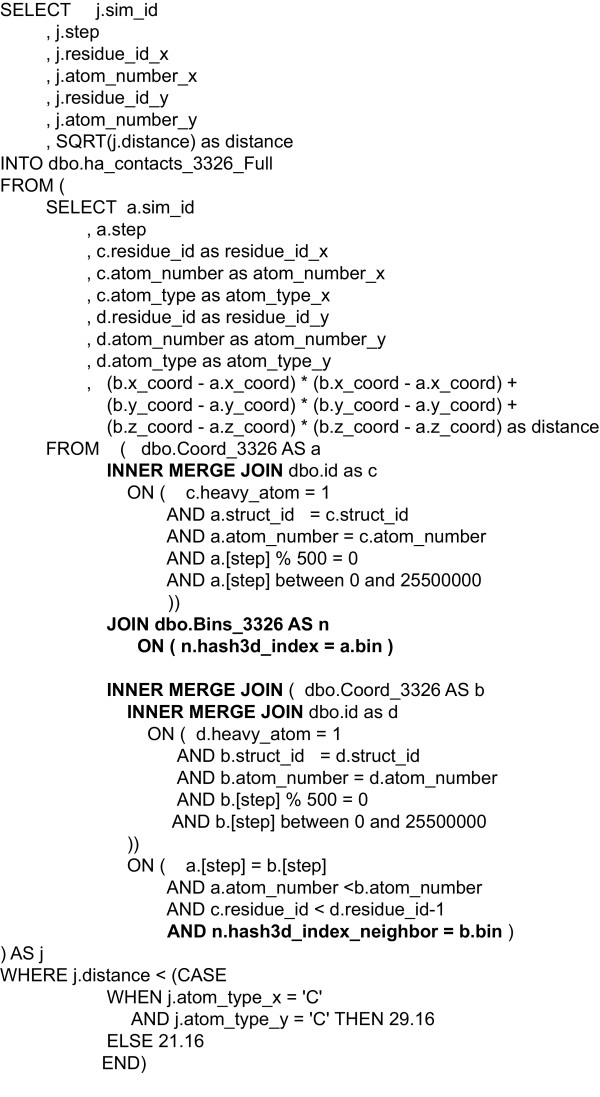
**Simplified heavy-atom contacts query**. The size of the join is reduced by applying two right associative join clauses, shown in **bold**. Right associative joins are a mechanism to control the order of join evaluation. In this case we insure that only the rows meeting satisfying the given predicates participate in the final self-join (i.e. heavy-atoms and only the first 1 ns of simulation time). The spatial-index join is shown in ***bold-italics***. This clause allows SQL to trim away most atoms outside the cutoff range without needing to perform the distance calculation, greatly reducing the number of operations as well as rows that would later be thrown away by the distance cutoff. Finally, MERGE joins are explicitly specified to avoid the optimizer choosing a HASH join for the coordinate table self-join.

To calculate contacts in SQL, an expensive self-join of the coordinate table must be used in addition to joins with structural data tables. A simplified version of this query is shown in Figure 7. Conditions in the JOIN clauses ensure that comparisons were made within the same frame (a.step = b.step) and with a granularity of 1 ps (a.step % 500 = 0 and b.step % 500 = 0). As distance is reflexive, we only calculated the distance from a heavy-atom in "a" to another in "b" where the atom index of a was less than b (a.atom_number < b.atom_number). We also excluded contacts in the same or adjacent residues (a.residue_id < b.residue_id - 1) as these are either through bond contacts or simply non-informative. Finally, the query only considered heavy-atoms, that is all non-hydrogen atoms (c.heavy_atom = 1 and d.heavy_atom = 1).

There are three supported join types in SQL Server: Hash, Merge, and Loop. Normally queries are expressed using only the keyword JOIN, leaving the optimizer free to choose the join type when an execution plan for a query is prepared. Join types are described in detail elsewhere [19]. The self-join of the coordinate table presented unique difficulty because of the size of the coordinate table. The optimizer will consistently choose a hash join, which will cause an expensive build of a temporary hash structure. In contrast, the merge join type does not require the temporary structure, and as the data are ordered based on the primary key, this approach is significantly faster.

We optimized the structure of the query with the use of two right associative joins [20] to cause early evaluation of the coordinate and atom ID table joins. We also pushed predicates directly into the join clauses. However, despite these optimizations a great deal of time was spent calculating distances for atoms that are outside the 5.4 Å distance of interest. These additional calculations added a significant performance burden, making it impractical to run this query over more than a handful of trajectories.

In our spatial indexing implementation we subdivided the periodic box used for simulation and divided it evenly into bins with dimensions of at least 5.4 Å on all axes. The bins were assigned consecutive numbers creating a one-dimensional hash. For our simulation data, the number of bins in a system does not exceed 2^32^-1 bins, so it is possible to represent the bin index as a SQL 32bit integer. Equation 1.1 was used to sort the atoms based on their coordinates *x, y*, and *z *into the bin array. Subsequently, Eq. 1.2 was used to calculate the bin index and this result was stored in the coordinate table, alongside the coordinates. The bins table was created for each simulation, in which each row is the list of 26 adjacent bins for a given bin index. This table was populated using a C# user defined function at the time the simulation coordinate data are loaded.(1.1)(1.2)

With the bins table in place, the contact query presented earlier can be modified slightly to filter coordinates considered using the bin column in the coordinate table. The modification is shown in **bold **(Figure 7). This simple join allows the query optimizer to quickly remove distance calculations based on a comparison of integer columns instead of projecting and transforming x, y, z from each half of the join. In this way, the bins table acted as a highly optimized spatial index.

### Table and index compression in the database

To investigate the effect of compression on database queries, we returned to the contacts query introduced earlier in this section, as it is a commonly used and computationally expensive query in trajectory analysis and reviewed performance data collected against all combinations of compression options across our sample set of 11 protein simulations. We also considered non-compressed and fully page compressed contact queries for the first 1 nanosecond that did not utilize the spatial indexing optimization.

### Database and System setup

Two Dell R710 servers each equipped with dual hex-core processors were used to collect run-time data. The base operating system is Windows Server 2008 Enterprise x64 R2 and the database engine used was SQL Server 2008 R2 Enterprise x64 R2. Detailed hardware and software configuration information is shown in Table 2.

**Table 2:** Detailed hardware and software configuration information

Hardware	Description
**Server**	Dell R710
**Processors**	Dual Intel Xeon X5650s (x64 Hex Core)
**Memory**	48 GB
**Storage**	H700 Integrated RAID SAS Disk Controller
**System Disks**	136 GB on two 15K RPM 150GB SAS disks, RAID 1 (Mirrored)
**Data Disks**	7,450 GB on six 7200 RPM 2TB SAS disks, RAID 0 (Striped)

**Software**	

**OS**	Windows Server 2008 R2 Enterprise x64
**Database**	SQL Server 2008 R2 Enterprise x64
**SQL**	Enabled for all CPUs
**SQL Memory**	Limited to 40,960 MB (8GB for OS)
**Anti-Virus**	Sophos Endpoint Security and Control, version 9

One database called hash3d-700 was created on each server and populated with a copy of coordinate trajectory tables and dimension tables from our primary data warehouse [7,21]. The base coordinate tables were then copied to additional tables, adding an additional suffix to indicate data and index compression settings. After all coordinate tables were created and populated, identical primary keys, constraints and indexes were applied. Tables were then compressed using **ALTER TABLE **statements. A script was run on all the coordinate table compression combinations to create contact tables. The size of each hash3d-700 database size was then adjusted upwards to 1.2 TB and the SQL Server process shutdown. Finally, the data and system partitions were defragmented with the **defrag.exe **to clean up file system fragmentation caused by auto-growth during loading.

Queries were run in SQL Server Management studio running on a remote machine with a connection to the test database server. Queries were executed with **SET STATISTICS IO ON **and **SET STATISTICS TIME ON **to capture logical and physical read statistics. To control for performance gains caused by data and/or query plan caching; and background write operations from result tables, a series of three system statements were executed prior to running the test query (Additional file 4, Figure S1). The **CHECKPOINT **statement insures that any dirty pages (such as those result rows written out by the previous query) are written to disk. The **FREESYSTEMCACHE **command eliminates any stored query or procedure plans. The **DROPCLEANBUFFERS **flushes out the current cache leaving it effectively cold, as though SQL Server had just started. During the collection of run-time data, access to both servers was restricted and only the query of interest was permitted to run.

We calculated the pairs of heavy-atom contacts for the 1st nanosecond of each simulation (1000 frames) and compared the execution times with and without spatial indexing. Queries were written in SQL and executed in MS SQL management studio as described in the above section. Heavy-atom contacts calculations were performed in triplicate for each simulation, ensuring the system cache was cleared between each run to obtain accurate performance statistics. Statistics were calculated using a two sample two-sided t-test for unequal variances.

We investigated 9 sets of compression options on both data and indices for each coordinate table for the 11 simulations in our test set. We recorded the extent of compression of each set of compression options compared with the non-compressed coordinate tables. We then ran an initial test of performance by investigating the execution time and disk input and output operations of the heavy-atom contacts query over the first nanosecond of the simulation. Subsequently, we examined the execution time of the heavy-atom contacts query over the full 51 ns (51,000 frames) native state trajectory for each of the proteins in our test set.

## Competing interests

The authors declare that they have no competing interests.

## Authors' contributions

RDT, AMS, DACB and VD designed the study, analyzed and interpreted the data and prepared the manuscript. RDT and AMS wrote the queries and carried out the run-time comparisons. DACB developed the original idea for the study. All authors read and approved the final manuscript.

## Supplementary Material

Additional file 1 **Table S1. Comparison of 1 ns contact query time with and without spatial indexing**. Statistics carried out using a two-sample t test with unequal variances - comparing contact query time with and without spatial indexing over 1 ns trajectories. Click here for file

Additional file 2 **Table S2. Comparison of 51 ns contact query time with/without spatial indexing on compressed and uncompressed tables**. Statistics carried out using a two-sample t test with unequal variances - comparing contact query time on compressed and uncompressed tables with and without spatial indexing over 51 ns trajectories. Click here for file

Additional file 3 **Table S3. Comparison of 51 ns contact query time with and without spatial indexing on compressed and uncompressed tables**. Tables showing the effect of spatial indexing on contact query time when the tables and compressed. Click here for file

Additional file 4 **Figure S1. SQL commands for clearing the system cache**. SQL commands for clearing the system cache. Click here for file
