# Supplementary material for: Implementation of 3D spatial indexing and compression in a large-scale molecular dynamics simulation database for rapid atomic contact detection
Source: BMC Bioinformatics. 2011 Aug 10;12:334. doi: 10.1186/1471-2105-12-334 (PMC3166946; doi:10.1186/1471-2105-12-334)
Supplement: Additional file 1 — Table S1. Comparison of 1 ns contact query time with and without spatial indexing. Statistics carried out using a two-sample t test with unequal variances - comparing contact query time with and without spatial indexing over 1 ns trajectories. [file 1471-2105-12-334-S1.DOC]

**Table S1. Comparison of 1 ns contact query time with and without spatial indexing.** Statistics carried out using a two-sample t test with unequal variances

| **PDB** | **SI** | **N** | **<Time>** | **SI** | **N** | **<Time>** | **Difference** | **% Difference** | **X faster** | **95% CI** | **p** |
| --- | --- | --- | --- | --- | --- | --- | --- | --- | --- | --- | --- |
| 2adr | 0 | 6 | 11.6 | 1 | 6 | 10 | 1.6 | 13.8 | 1.2 | (-5.34, 8.55) | 0.31 |
| 1nr2 | 0 | 6 | 29.3 | 1 | 6 | 16.7 | 12.6 | 43.0 | 1.8 | (11.18, 14.10) | 0 |
| 1okt | 0 | 6 | 56.1 | 1 | 6 | 23.7 | 32.5 | 57.8 | 2.4 | (30.85, 34.09) | 0 |
| 2tgi | 0 | 6 | 89.7 | 1 | 6 | 28.2 | 61.5 | 68.6 | 3.2 | (56.53, 66.38) | 0 |
| 1d0n | 0 | 6 | 127.9 | 1 | 6 | 32.6 | 95.3 | 74.5 | 3.9 | (90.63, 99.99) | 0 |
| 1bp5 | 0 | 6 | 180.2 | 1 | 6 | 37.5 | 142.7 | 79.2 | 4.8 | (138.40, 147.03) | 0 |
| 1hgu | 0 | 6 | 256.4 | 1 | 6 | 42.4 | 214 | 83.5 | 6.0 | (210.81, 217.25) | 0 |
| 1p88 | 0 | 6 | 294.4 | 1 | 6 | 51.7 | 242.7 | 82.4 | 5.7 | (236.38, 248.92) | 0 |
| 1fzw | 0 | 6 | 578.3 | 1 | 6 | 73.4 | 504.8 | 87.3 | 7.9 | (498.92, 510.77) | 0 |
| 1qaz | 0 | 6 | 860.4 | 1 | 6 | 95.1 | 765.3 | 88.9 | 9.0 | (753.80, 776.85) | 0 |
| 1ehe | 0 | 6 | 1091.8 | 1 | 6 | 105.6 | 986.2 | 90.3 | 10.3 | (974.65, 997.79) | 0 |

PDB= pdb code for the representative used. SI = spatial index. N = number of samples. <time> average execution time in seconds. Difference = average difference in execution time. % Difference is calculated as ((<Time_noSI_> - <Time_SI_>) / <Time_noSI_> )X 100. X faster is calculated as <Time_noSI_>/<Time_SI_> is an indicator of how many times faster the spatial indexing is over non spatial indexed tables. 95% CI= 95% confidence interval, p= p-value
