# Supplementary material for: Implementation of 3D spatial indexing and compression in a large-scale molecular dynamics simulation database for rapid atomic contact detection
Source: BMC Bioinformatics. 2011 Aug 10;12:334. doi: 10.1186/1471-2105-12-334 (PMC3166946; doi:10.1186/1471-2105-12-334)
Supplement: Additional file 2 — Table S2. Comparison of 51 ns contact query time with/without spatial indexing on compressed and uncompressed tables. Statistics carried out using a two-sample t test with unequal variances - comparing contact query time on compressed and uncompressed tables with and without spatial indexing over 51 ns trajectories. [file 1471-2105-12-334-S2.DOC]

**Table S2a. Comparison of 51 ns contact query time without spatial indexing on compressed and uncompressed tables.** Statistics carried out using a two-sample t test with unequal variances

| **PDB** | **Comp-1** | **N** | **<Time>** | **Comp-2** | **N** | **<Time>** | **Difference** | **95% CI** | **p** |
| --- | --- | --- | --- | --- | --- | --- | --- | --- | --- |
| 2adr | NN | 3 | 482.2 | PP | 3 | 486.4 | -4.2 | (-26.19, 17.82) | 0.69 |
| 1nr2 | NN | 3 | 1592.9 | PP | 3 | 1608.7 | -15.8 | (-59.20, 27.58) | 0.82 |
| 1okt | NN | 3 | 2866.5 | PP | 3 | 2918.4 | -51.9 | (-244.00, 140.28) | 0.78 |
| 2tgi | NN | 3 | 4438.3 | PP | 3 | 4464.8 | -26.4 | (-140.83, 87.96) | 0.72 |
| 1d0n | NN | 3 | 6256.6 | PP | 3 | 6252.9 | 3.7 | (-149.68, 157.05) | 0.47 |
| 1bp5 | NN | 3 | 8922.4 | PP | 3 | 9113.9 | -191.5 | (-1058.00, 674.98) | 0.77 |
| 1hgu | NN | 3 | 12578.6 | PP | 3 | 12634 | -55.4 | (-472.06, 361.28) | 0.68 |
| 1p88 | NN | 3 | 14310.3 | PP | 3 | 14358.3 | -48 | (-326.01, 230.06) | 0.67 |
| 1fzw | NN | 6 | 27756.2 | PP | 6 | 30548.9 | -2792.7 | (-9959.08, 4373.64) | 0.82 |
| 1qaz | NN | 3 | 43456.2 | PP | 5 | 42074.9 | 1381.3 | (-5140.15, 7902.75) | 0.29 |
| 1ehe | NN | 3 | 52838.7 | PP | 3 | 53669.9 | -831.3 | (-2309.40, 646.87) | 0.93 |

**Table S2b. Comparison of 51 ns contact query time with spatial indexing on compressed and uncompressed tables.** Statistics carried out using a two-sample t test with unequal variances

| **PDB** | **Comp-1** | **N** | **<Time>** | **Comp-2** | **N** | **<time>** | **difference** | **95% CI** | **p** |
| --- | --- | --- | --- | --- | --- | --- | --- | --- | --- |
| 2adr | NN | 6 | 1064.1 | PP | 6 | 963.7 | 100.5 | (-61.07, 262.03) | 0.09 |
| 1nr2 | NN | 6 | 1632.1 | PP | 6 | 1545.8 | 86.3 | (-260.31, 433.00) | 0.28 |
| 1okt | NN | 6 | 1977.1 | PP | 6 | 2065.1 | -88 | (-294.22, 118.21) | 0.82 |
| 2tgi | NN | 6 | 2508.5 | PP | 6 | 2732.8 | -224.3 | (-480.51, 31.96) | 0.96 |
| 1d0n | NN | 6 | 2854.3 | PP | 6 | 2833.8 | 20.4 | (-272.43, 313.32) | 0.44 |
| 1bp5 | NN | 6 | 3738 | PP | 6 | 3790 | -52 | (-413.02, 309.00) | 0.62 |
| 1hgu | NN | 6 | 4343.3 | PP | 6 | 4395.7 | -52.4 | (-515.52, 410.65) | 0.60 |
| 1p88 | NN | 6 | 4688.2 | PP | 6 | 4820.8 | -132.6 | (-502.85, 237.61) | 0.78 |
| 1fzw | NN | 6 | 6821 | PP | 6 | 6868.9 | -48 | (-575.63, 479.66) | 0.58 |
| 1qaz | NN | 6 | 8751 | PP | 6 | 8390.1 | 360.9 | (-619.33, 1341.05) | 0.22 |
| 1ehe | NN | 6 | 10049.6 | PP | 6 | 9941.4 | 108.1 | (-1004.09, 1220.36) | 0.42 |

PDB= pdb code for the representative protein used. Comp#=Compression combination applied where NN indicates no compression applied to either data or index and PP indicates page level compression is applied to the data and the index. SI = spatial index. N = number of samples. <time> average execution time in seconds. difference = average difference in execution time.  Difference = average difference in execution time . 95% CI= 95% confidence interval. p= p-value
