# Supplementary material for: Implementation of 3D spatial indexing and compression in a large-scale molecular dynamics simulation database for rapid atomic contact detection
Source: BMC Bioinformatics. 2011 Aug 10;12:334. doi: 10.1186/1471-2105-12-334 (PMC3166946; doi:10.1186/1471-2105-12-334)
Supplement: Additional file 3 — Table S3. Comparison of 51 ns contact query time with and without spatial indexing on compressed and uncompressed tables. Tables showing the effect of spatial indexing on contact query time when the tables and compressed. [file 1471-2105-12-334-S3.DOC]

**Table S3a. Comparison of 51 ns contact query time with and without spatial indexing on uncompressed tables**

| **Pdb** | **Compression** | **SI** | **N** | **<time>** | **SI** | **N** | **<time>** | **%difference** | **X faster** |
| --- | --- | --- | --- | --- | --- | --- | --- | --- | --- |
| 2adr | NN | 0 | 3 | 482.2 | 1 | 6 | 1064.1 | -120.7 | 0.5 |
| 1nr2 | NN | 0 | 3 | 1592.9 | 1 | 6 | 1632.1 | -2.5 | 1.0 |
| 1okt | NN | 0 | 3 | 2866.5 | 1 | 6 | 1977.1 | 31.0 | 1.4 |
| 2tgi | NN | 0 | 3 | 4438.3 | 1 | 6 | 2508.5 | 43.5 | 1.8 |
| 1d0n | NN | 0 | 3 | 6256.6 | 1 | 6 | 2854.3 | 54.4 | 2.2 |
| 1bp5 | NN | 0 | 3 | 8922.4 | 1 | 6 | 3738 | 58.1 | 2.4 |
| 1hgu | NN | 0 | 3 | 12578.6 | 1 | 6 | 4343.3 | 65.5 | 2.9 |
| 1p88 | NN | 0 | 3 | 14310.3 | 1 | 6 | 4688.2 | 67.2 | 3.1 |
| 1fzw | NN | 0 | 3 | 27756.2 | 1 | 6 | 6821 | 75.4 | 4.1 |
| 1qaz | NN | 0 | 3 | 43456.2 | 1 | 6 | 8751 | 79.9 | 5.0 |
| 1ehe | NN | 0 | 3 | 52838.7 | 1 | 6 | 10049.6 | 81.0 | 5.3 |

**Table S3b. Comparison of 51 ns contact query time with and without spatial indexing on compressed tables**

| **Pdb** | **Compression** | **SI** | **N** | **<time>** | **SI** | **N** | **<time>** | **%difference** | **X faster** |
| --- | --- | --- | --- | --- | --- | --- | --- | --- | --- |
| 2adr | PP | 0 | 3 | 486.4 | 1 | 6 | 963.7 | -98.1 | 0.5 |
| 1nr2 | PP | 0 | 3 | 1608.7 | 1 | 6 | 1545.8 | 3.9 | 1.0 |
| 1okt | PP | 0 | 3 | 2918.4 | 1 | 6 | 2065.1 | 29.2 | 1.4 |
| 2tgi | PP | 0 | 3 | 4464.8 | 1 | 6 | 2732.8 | 38.8 | 1.6 |
| 1d0n | PP | 0 | 3 | 6252.9 | 1 | 6 | 2833.8 | 54.7 | 2.2 |
| 1bp5 | PP | 0 | 3 | 9113.9 | 1 | 6 | 3790 | 58.4 | 2.4 |
| 1hgu | PP | 0 | 3 | 12634 | 1 | 6 | 4395.7 | 65.2 | 2.9 |
| 1p88 | PP | 0 | 3 | 14358.3 | 1 | 6 | 4820.8 | 66.4 | 3.0 |
| 1fzw | PP | 0 | 6 | 30548.9 | 1 | 6 | 6868.9 | 77.5 | 4.4 |
| 1qaz | PP | 0 | 5 | 42074.9 | 1 | 6 | 8390.1 | 80.1 | 5.0 |
| 1ehe | PP | 0 | 3 | 53669.9 | 1 | 6 | 9941.4 | 81.5 | 5.4 |

PDB= pdb code for the representative used. Compression= Compression combination applied where NN indicates no compression applied to either data or index and PP indicates page level compression is applied to the data and the index. SI = spatial index. N = number of samples. <time> = average execution time in seconds. Difference = average difference in execution time . % Difference is calculated as ((<Time_noSI_> - <Time_SI_>) / <Time_noSI_> )X 100. X faster is calculated as <Time_noSI_>/<Time_SI_> is an indicator of how many times faster the spatial indexing is over non spatial indexed tables.
