# Supplementary material for: Implementation of 3D spatial indexing and compression in a large-scale molecular dynamics simulation database for rapid atomic contact detection
Source: BMC Bioinformatics. 2011 Aug 10;12:334. doi: 10.1186/1471-2105-12-334 (PMC3166946; doi:10.1186/1471-2105-12-334)
Supplement: Additional file 4 — Figure S1. SQL commands for clearing the system cache. SQL commands for clearing the system cache. [file 1471-2105-12-334-S4.DOCX]

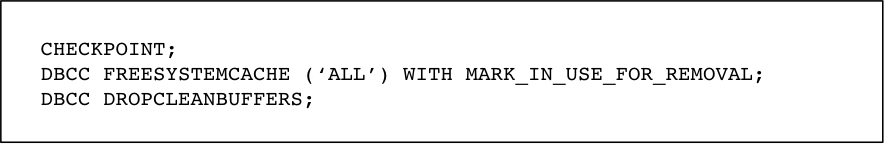


**Figure S1. SQL commands for clearing the system cache.** These commands were run after each test calculation to ensure there were no cached data.
